# Ultrasonic Emulsification of Severe Mitral Annular Calcification to Enable Mitral Valve Repair

**DOI:** 10.1177/15569845231225672

**Published:** 2024-01-29

**Authors:** Djalal Fakim, Manuel Cervetti, A. Dave Nagpal, Aashish Goela, Michael W. A. Chu

**Affiliations:** 1 Division of Cardiac Surgery, Department of Surgery, Western University, London, ON, Canada; 2 Department of Medical Imaging, Western University, London, ON, Canada

## Introduction

Mitral annular calcification (MAC) increases surgical complexity and is associated with increased perioperative risks.^1–4^ Treatment of mitral disease with MAC is challenging, often requiring mitral valve replacement (MVR).^4^ Mitral annular decalcification to enable mitral valve repair (MVr) requires preserving leaflet integrity and sufficient calcium debulking to enable annular remodeling and unrestricted leaflet motion without disrupting the atrioventricular groove. Care must be exercised to ensure that calcium fragmentation and debridement are associated with minimal embolic risks. We describe an innovative approach using ultrasonic emulsification and aspiration to decalcify the mitral annulus to achieve successful MVr in a patient with severe, degenerative MAC.

## Case Report

**Fig. 1.**
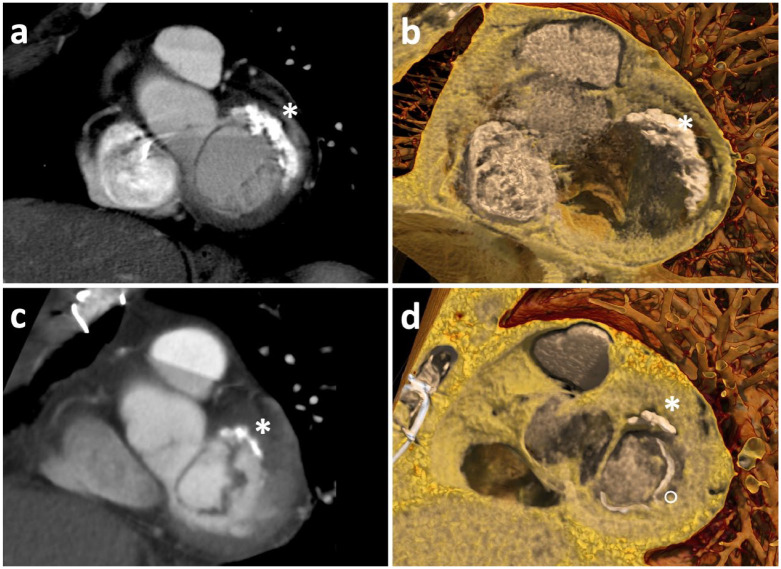
(a) Preoperative CT of the heart demonstrating severe MAC (*). (b) Global illumination rendering of severe MAC (*) in preoperative CT of the heart. (c) Postoperative CT of the heart demonstrating significant reduction in MAC (*). (d) Global illumination rendering of reduced MAC in postoperative CT of the heart demonstrating significant MAC reduction (*) and annuloplasty band (°). CT, computed tomography; MAC, mitral annulus calcification.

A 53-year-old woman with known severe mitral valve insufficiency, gastroesophageal reflux disease, hypothyroidism, dyspepsia, and previous breast reduction surgery presented with increasing shortness of breath, in keeping with New York Heart Association Class III symptoms. Echocardiography demonstrated bileaflet prolapse with severe mitral regurgitation and pulmonary vein flow reversal, with normal ejection fraction. Transesophageal echocardiography demonstrated severe mitral regurgitation and severe MAC, with a potentially repairable valve. Computed tomography confirmed the extensive posterior MAC (Fig. 1a) and demonstrated no significant coronary disease with a right dominant coronary artery. Our a priori plan was to perform a posterior bar decalcification with sharp dissection and complex mitral repair. Traditionally, we usually excise the posterior bar calcification with a combination of a scalpel and electrocautery; however, we decided to trial calcific emulsification with the Sonopet Qi ultrasonic aspirator (Sonopet) system (Stryker, Kalamazoo, MI, USA). A minimally invasive approach was initially planned, but the decision was made prior to incision to change to a sternotomy to accommodate the shorter length of the Sonopet tool.

**Fig. 2.**
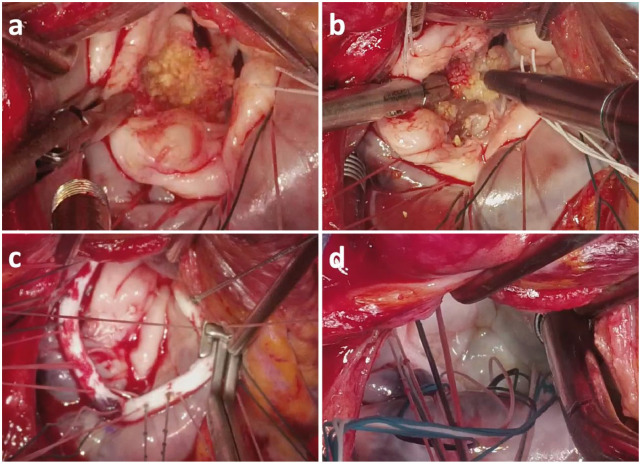
Intraoperative images demonstrating (a) severe MAC, (b) use of Sonopet Qi Ultrasonic Aspirator (Stryker, Kalamazoo, MI, USA) to emulsify MAC, (c) 36 mm Cosgrove band annuloplasty (Edwards Lifesciences, Irvine, CA, USA), and (d) final result. MAC, mitral annular calcification.

At operation, the patient underwent midline sternotomy and standard central cannulation. A transverse left atriotomy approach was used, and selected annuloplasty sutures were placed to optimize surgical exposure. The extensive MAC was easily identified subtending the base of A1, lateral commissure, and P1 and P2 segments and was restricting diastolic motion of the posterior leaflet (PL). The MAC was adherent to the base of P1 and P2, where we had concerns of sacrificing too much of the PL with conventional decalcification methods. We began targeted calcific emulsification below the mitral valve, beneath P1 and P2 (Fig. 2b, Supplemental Video). A quadrangular resection at the lateral aspect of P2 was performed to provide better exposure and enable further decalcification of the entire annulus. Interestingly, the Sonopet allows fine and detailed emulsification such that it can allow enough calcium removal to enable a pliable leaflet and annulus without necessarily requiring complete removal at the level of the atrioventricular groove. The left ventricle was irrigated extensively to capture any calcium debris. A sliding plasty was performed, and the quadrangular resection was reapproximated. The lateral commissure was advanced with a commissuroplasty suture. A 36 mm Cosgrove band annuloplasty (Edwards Lifesciences, Irvine, CA, USA) was selected and implanted with interrupted braided suture (Fig. 2c). The mitral valve was found to be competent on saline test. The patient was easily weaned from cardiopulmonary bypass, and intraoperative transesophageal echocardiogram demonstrated no residual mitral insufficiency with a mean and peak gradient of 3 and 11 mm Hg, respectively, with a height of coaptation of 9 mm. The patient had an uncomplicated postoperative recovery and was discharged home on the ninth postoperative day. At 3 months postoperatively, the patient was well. Echocardiography confirmed a normally functioning mitral repair with no mitral insufficiency; a mean and peak gradient of 3 and 9 mm Hg, respectively; and good biventricular function. Follow-up computed tomography of the heart confirmed the significant reduction in MAC with near complete decalcification at the annular level (Fig. 1c–d).

## Discussion

Contemporary surgical approaches to MAC include suturing through or around the MAC, implanting a transcatheter prosthesis within the MAC, or decalcification of the MAC. Most of these approaches result in MVR.^1^ The “respect” method allows the implantation of the prosthesis overtop the calcium bar, which can lead to poor annular sealing and significant paravalvular leak (PVL)^1^ or may require undersizing of the mitral prosthesis, resulting in compromised hemodynamics. The “resect” approach involves en bloc decalcification and annular reconstruction and requires advanced technical expertise with longer cardiopulmonary bypass and cross-clamp times. The “resect” technique allows for a larger MVR prosthesis with improved sealing with decreased PVL; however, it risks weakening the mitral annulus and atrioventricular groove, leading to potential disruption and high operative mortality.^1^ Although MAC has been associated with increased perioperative mortality in single-center series, a recent meta-analysis suggested no significant differences in perioperative mortality in patients with and without MAC.^2,3^ In selected degenerative cases, en bloc calcific bar resection can allow complex mitral repair, which theoretically may be associated with less perioperative risks than MVR in patients with severe MAC. Traditional techniques of calcific bar resection require sharp dissection of the entire calcific block, which may increase the risks of atrioventricular groove disruption and can be associated with significant calcium fragmentation and embolic risks during debridement.

Ultrasonic emulsification with the Sonopet device enables targeted calcific fragmentation and aspiration through cavitation, a process involving the emulsification of hydrous tissue (such as calcium, tumor, or fat) utilizing microscopic bubbles. Anhydrous tissues, such as blood vessels, muscles, nerves, and tendons, are unaffected by cavitation. As such, the Sonopet allows for more accurate debridement of the MAC compared with the current standard of care with scalpel or cautery, perhaps without increasing the risk of damage to the left ventricle and atrioventricular groove. Increased accuracy allows for sculpting of the MAC resection, optimizing annular mobility and remodeling while preserving leaflet tissue and leaflet mobility. Importantly, the ultrasonic emulsification and aspiration of MAC minimizes embolic risks. We generally start with the following Sonopet settings: power 80%, suction 70%, and irrigation 20%, and increase the power for very hard calcification. In terms of safety, ultrasonic emulsification could potentially cause collateral tissue damage if the tip of the device is not controlled accurately; however, we do feel that it may be safer than more traditional methods of sharp decalcification with a scalpel or electrocautery as the emulsification process targets the calcium while sparing the muscle of the atrium and ventricle. As we remain in the early learning phase with this device, cautious use and vigilance will remain important. An early report from Brescia et al. in 15 patients who underwent MVR with emulsification of MAC using the Sonopet device demonstrated 0% mortality and 0% stroke in comparison with the non-MAC emulsification group, with rates of 10% and 17%, respectively, although there were no significant differences between the groups when accounting for the total historical series of MVR with MAC.^4^ In that series, median hospital length of stay, mean gradient at last echocardiogram, and rate of reoperation did not differ by group.^4^

This case demonstrates the utility of MAC ultrasonic emulsification and aspiration to enable successful MVr in a patient with severe MAC. This novel surgical technique should be considered in the surgical armamentarium to treat MAC, which may aid in reducing perioperative risks and in some cases enable complex valve repair.

## References

[1] Alexis SL Malik AH El-Eshmawi A , et al. Surgical and transcatheter mitral valve replacement in mitral annular calcification: a systematic review. J Am Heart Assoc 2021; 10: e018514. 10.1161/JAHA.120.018514 PMC8174336 33728929

[2] El-Eshmawi A Alexis SL Sengupta A , et al. Surgical management of mitral annular calcification. Curr Opin Cardiol 2020; 35: 107–115. 31895243 10.1097/HCO.0000000000000718

[3] Ribeiro RVP Yanagawa B Légaré JF , et al. Clinical outcomes of mitral valve intervention in patients with mitral annular calcification: a systematic review and meta-analysis. J Card Surg 2020; 35: 66–74. 31692124 10.1111/jocs.14325

[4] Brescia AA Rosenbloom LM Watt TMF , et al. Ultrasonic emulsification of severe mitral annular calcification during mitral valve replacement. Ann Thorac Surg 2022; 113: 2092–2096. 34990573 10.1016/j.athoracsur.2021.11.066

